# Efficient mutation screening for cervical cancers from circulating tumor DNA in blood

**DOI:** 10.1186/s12885-020-07161-0

**Published:** 2020-07-27

**Authors:** Sun-Young Lee, Dong-Kyu Chae, Sung-Hun Lee, Yohan Lim, Jahyun An, Chang Hoon Chae, Byung Chul Kim, Jong Bhak, Dan Bolser, Dong-Hyu Cho

**Affiliations:** 1 Department of Radiation Oncology, Jeonbuk National University Hospital-Jeonbuk National University Medical School, Jeonju, Jeonbuk Republic of Korea; 2 Research Institute of Clinical Medicine of Jeonbuk National University-Biomedical Research Institute of Jeonbuk National University Hospital, Jeonju, Republic of Korea; 3 Clinomics Inc, Suwon, 16229 Republic of Korea; 4 Department of Biophysics and Radiation Biology, Lab of Nanochemistry, Semmelweis University, Budapest, Hungary; 5 KOGIC, UNIST, Ulsan, 44919 Republic of Korea; 6 Geromics LTD, Cambridge, CB1 1AH UK; 7 Department of Obstetrics and Gynecology, Jeonbuk National University Hospital-Jeonbuk National University Medical School, Jeonju, Jeonbuk Republic of Korea

**Keywords:** Cervical cancer, Next-generation-sequencing, Circulating tumor DNA, Cancer panel, Genomic alteration

## Abstract

**Background:**

Early diagnosis and continuous monitoring are necessary for an efficient management of cervical cancers (CC). Liquid biopsy, such as detecting circulating tumor DNA (ctDNA) from blood, is a simple, non-invasive method for testing and monitoring cancer markers. However, tumor-specific alterations in ctDNA have not been extensively investigated or compared to other circulating biomarkers in the diagnosis and monitoring of the CC. Therfore, Next-generation sequencing (NGS) analysis with blood samples can be a new approach for highly accurate diagnosis and monitoring of the CC.

**Method:**

Using a bioinformatics approach, we designed a panel of 24 genes associated with CC to detect and characterize patterns of somatic single-nucleotide variations, indels, and copy number variations. Our NGS CC panel covers most of the genes in The Cancer Genome Atlas (TCGA) as well as additional cancer driver and tumor suppressor genes. We profiled the variants in ctDNA from 24 CC patients who were being treated with systemic chemotherapy and local radiotherapy at the Jeonbuk National University Hospital, Korea.

**Result:**

Eighteen out of 24 genes in our NGS CC panel had mutations across the 24 CC patients, including somatic alterations of mutated genes (*ZFHX3–*83%, *KMT2C-*79%*, KMT2D-*79%, NSD1–67%, *ATM-*38% and *RNF213*–27%). We demonstrated that the *RNF213* mutation could be used potentially used as a monitoring marker for response to chemo- and radiotherapy.

**Conclusion:**

We developed our NGS CC panel and demostrated that our NGS panel can be useful for the diagnosis and monitoring of the CC, since the panel detected the common somatic variations in CC patients and we observed how these genetic variations change according to the treatment pattern of the patient.

## Background

Cervical cancer (CC) is the third most frequently diagnosed cancer and the fourth most common cause of cancer-related death among women worldwide, particularly in developing countries [1]. Although the development of a screening method for human papillomavirus (HPV)-based diagnosis for CC and HPV vaccination have lowered the incidence and death rate, this cancer still remains among the most common causes of cancer-related death in women [2]. High-risk human papillomavirus (HR-HPV), which is difficult to eradicate by the host immune system, infects the epithelial layer of the cutaneous and mucosal surfaces [3, 4]. The mechanism of HR-HPV infection is an important carcinogenic factor that increases the risk of CC development over time. It has been reported that 15–30% of patients with early-stage CC experience recurrences after surgical operation, and half of those who previously had recurrent cancer show a higher risk of another recurrent cancer within 3 years after primary treatment. Thus, it is recommended that patients visit clinics for check-ups every 3–4 months for the first 2 years, and every 6–12 months for the next 3–5 years after initial treatment to monitor the recurrence of CC [5]. During a check-up for cancer recurrence, cervical cytology, measurement of squamous cell carcinoma antigen and CA-125 in the blood, and medical imaging techniques such as computed tomography, magnetic resonance imaging, and positron emission spectroscopy are performed. However, the cervical cytology and the blood tests are limited by their low sensitivity and specificity. Medical imaging techniques can be employed to improve the detection of cancer recurrence, but they are costly and involve radiation exposure. Recent liquid biopsy studies showed that cell-free DNA (cfDNA), which originates from the apoptosis and necrosis of normal and tumor cells may be valuable for monitoring tumor behaviors and treatment responses [6–8]. The detection of HPV16 and HPV18 DNA or alterations in the cfDNA of patients with CC patients is used as biomarkers for recurrence monitoring [9–11].

For these reasons, we built a custom NGS panel consisting of 24 genes related to gynecological cancers. In this study, we assessed the clinical utility of analyzing gene mutations in CC patients who have a medical history of chemotherapy and radiotherapy, profiling the genetic variations in cfDNA from 24 patients. As a result, we were able to obtain the mutational variations in ctDNA and observe their patterns over time, which can be used to detect the phases of CC, monitor the tumor status, and predict therapeutic responses.

## Methods

### Samples and clinical data

A total of 24 CC patients were enrolled in a prospective cohort at the Jeonbuk National University Hospital. All subjects provided an informed consent to participate in the study, and all clinical specimens were collected with approval from the institutional review board (IRB No. CUH2017–04–018-001) and ethics committee of Jeonbuk National University Hospital. Total of 7 ml of whole peripheral blood was collected into EDTA tubes from each patient approximately 1 week before chemotherapy. Genetic variations were analyzed using our NGS CC panel. For each patient, mutations were characterized from both cfDNA, plasma, and peripheral blood mononuclear cells (PBMCs).

### Sample preparation

PBMCs were isolated from 7 ml of whole blood by density gradient centrifugation in Ficoll-Paque™ PLUS (GE Healthcare, Little Chalfont, UK). We extracted cfDNA from isolated plasma using the QIAamp Circulating Nucleic Acid Kit (Qiagen, Hilden, Germany) according to the manufacturer’s instructions. The extracted DNA was quantified using a Qubit 3.0 fluorometer (Invitrogen, Carlsbad, CA, USA). We analyzed the quality of cfDNA using the 2100 Bioanalyzer (Agilent Technologies, Santa Clara, CA, USA) to detect genomic DNA contamination.

### Next-generation-sequencing

A total of 2265 amplicons were designed in two primer pools to capture the targeted regions. Amplicon size was designed to be 125–140 base pairs (bp), and the total number of bases covered by the amplicons was 169.34 kb. A total of 10 ng of cfDNA and PBMC-derived DNA was used for library construction. Library preparation was performed using an Ion Ampliseq Library Kit 2.0 (Thermo Fisher Scientific, Waltham, MA, USA) according to the manufacturer’s instructions. We used the Ion Express Barcode Adaptors Kit (Thermo Fisher Scientific) for sample multiplexing, and libraries were purified using the Agencourt AMPure XP reagent (Beckman Coulter, Brea, CA, USA). Libraries were quantified using the Qubit 3.0 fluorometer and 2100 Bioanalyzer. Template preparation of the libraries was performed using the Ion Chef Instrument (Thermo Fisher Scientific) with an Ion 540 Chef Kit (Thermo Fisher Scientific). Multiplexed templates were subjected to sequencing on the Ion S5 XL system (Thermo Fisher Scientific). PBMCs were evaluated to analyze somatic mutations and exclude germline mutations. Our panel can detect 0.1% tumor mutated cfDNA to normal cfDNA (range of read depths from 1000x to 3902x with a median read depth of 1554x). However, given the percentage of mutated tumor present in cfDNA, we set cut-off value of variation as 1%.

### Variant analysis

The human genome sequence hg19 was used as a reference for variant calling. Sequence and data analyses were performed using Torrent Suite software (5.8.0). Sequencing coverage analysis was performed using coverage Analysis (5.8.0.1) plugins, and VCF files were generated using the variantCaller (5.8.0.19) plugins. Annotations of the variants were obtained using Ion Reporter (5.10.2.0) software. To filter out the potential sequencing background noise, we excluded common Korean single-nucleotide variations, which are from KoVariome (http://variome.net). whole genome sequence database of 50 healthy unrelated Korean individuals [12, 13] and patient specific normal variants detected in PMBCs. After filtering (described above), the resulting cfDNA somatic mutations were annotated using the COSMIC database (https://cancer.sanger.ac.uk/cosmic) for comparison with previously reported variants.

## Results

### CC targeted NGS panel

Generally, liquid biopsies accompanying genomic analysis alone cannot identify all the features of the primary tumor. However, genetic alterations occurring in cancer patients must reflect cancer type specific mutations. Using our CC-targeted NGS panel, we first tried to detect any CC specific genetic variation in the patients. Then we sought to check the general mutation patterns of usual oncogenes, such as *PIK3CA* or *TP53*.

Based on TCGA database, we designed NGS CC-panel (Table 1) consists of 24 genes that are known to occur in gynecologic cancer at a high frequency. It contains 67% of genes that have been previously reported as significantly mutated in many cancers (SMGs - *PIK3CA*, *EP300*, *FBXW7*, *HLA-B*, *PTEN*, *NFE2L2*, *ARID1A*, *KRAS*, and *MAPK* [14]). This panel also covers 55% of the top 20, and 80% of the top ten genes detected in CC-related tumor tissue, according to the COSMIC data. The included genes are *PIK3CA*, *KRAS*, *TP53*, *PTEN*, *KMT2C*, *FBXW7*, *KMT2D*, *EP300*, *ARID1A*, *FAT1*, and *ZFHX3*. The other genes in our panel are related to tumor suppressor activity [15–17].

**Table 1:** Gene list in customized CC panel

***ARID1A***	***ATM***	***BCOR***	***CHD4***	***CTCF***
***CTNNB1***	***EP300***	***FAT1***	***FAT4***	***FBXW7***
***FGFR2***	***KMT2C***	***KMT2D***	***KRAS***	***NSD1***
***OR14K1***	***PIK3CA***	***PIK3R1***	***POLE***	***PTEN***
***RNF213***	***TP53***	***TRRAP***	***ZFHX3***	

### Evaluation of panel through reference materials

**Fig. 1:**
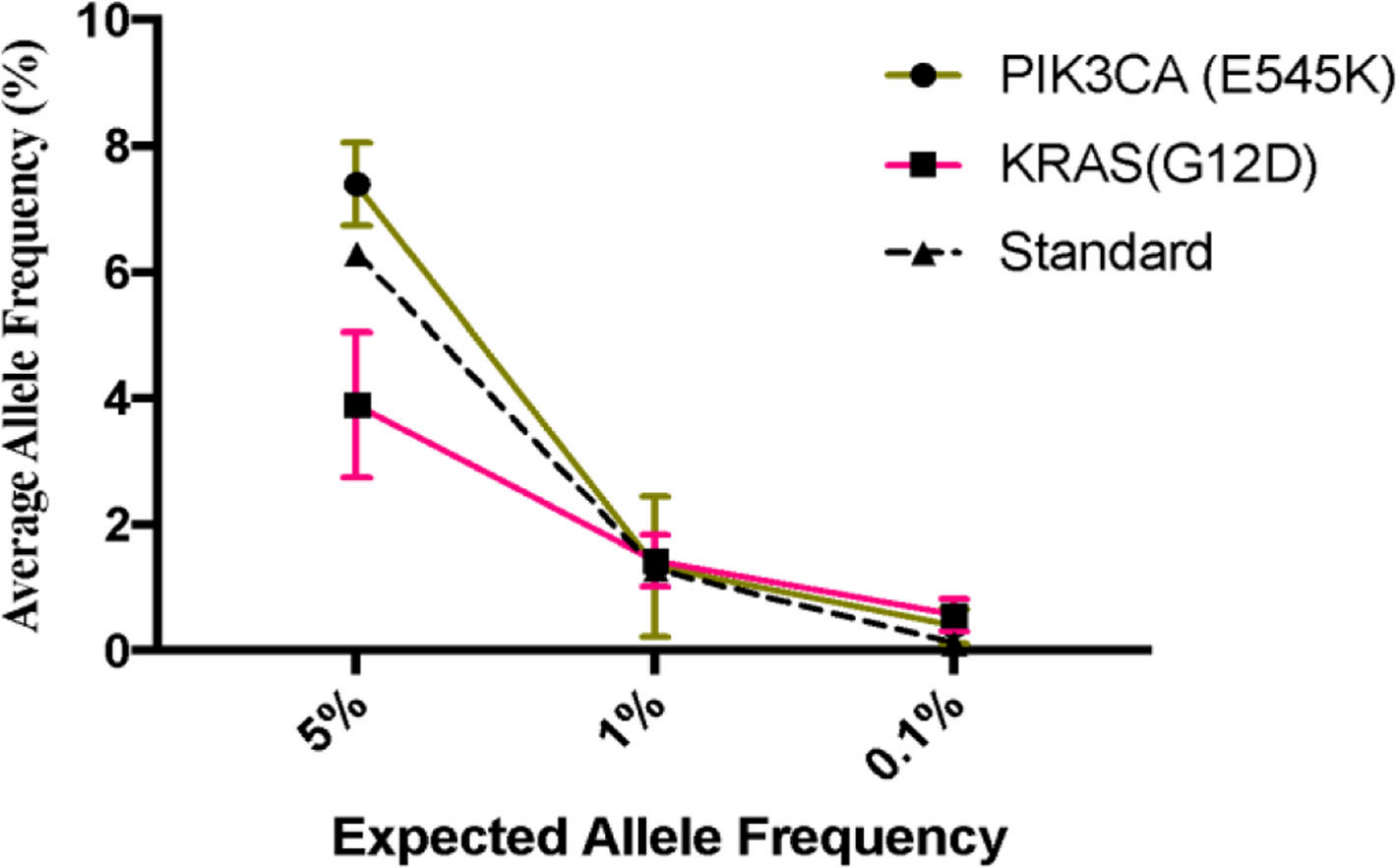
Verification with standard material. Based on comparisons using standard substances, NGS analysis confirmed the allele frequency of PIK3CA(E545K) and KRAS(G12D) with 1% of accuracy

We verified the NGS panel with standard materials to determine its sensitivity. The standard material (Horizon Discovery, Cambridge, UK) contains mutations in *PIK3CA*(E545K) and *KRAS*(G12D) genes. For accuracy, the sequencing was performed under the same conditions as the patient samples (10 ng input giving 1000-fold coverage). As a result of using 5% Multiplex I cfDNA Reference Standard, 6.3% variation was detected. Subsequently, 1.3 and 0.13% of allele frequency were identified under the utilization of 1 and 0.1% of standard material (Fig. 1). In addition, the average variations for *PIKC3A* and *KRAS* were 1.33 and 1.6%, respectively, based on the verification using 1% standard material. The allele frequencies of *PIK3CA* and *KRAS* were 0.27 and 0.4%, respectively. All samples were evaluated in triplicate, and the detection of standards confirmed that our panel was sensitive enough to detect 0.1% of genetic variation. To verify the sensitivity and specificity of this panel, the gene mutations were identified by digital droplet PCR, which indicated that the sensitivity for the *PIK3CA* gene was 88.9% and specificity was 100%. The result of the ddPCR also confirmed 100% sensitivity and specificity in the detection of the *KRAS* gene (Table 2).

**Table 2:** Verification of NGS panel using ddPCR as a gold standard

	Positive	Negative	Total	Sensitivity	Specificity
PIK3CA(E545K)					
Positive	8	1	9	**88.9%**	**100%**
Negative	0	3	3		
Total	8	4	12		
KRAS(G12D)					
Positive	9	0	9	**100%**	**100%**
Negative	0	3	3		
Total	9	3	12		

### Characteristics of patients

Twenty-four patients with CC were enrolled in this study. The criteria for enrollment was not case-controlled; therefore, patients were not specifically classified by cancer stage or histology, which could have introduced bias. The clinical and histopathological characteristics of these patients are summarized in Table 3. Blood samples were collected from the patients approximately 1 week prior to primary treatment. Genetic alteration was monitored in four patients who agreed to provide blood during the treatment, and their blood samples were drawn three times for the prognosis prediction. The median age in our cohort was 61 years and 25% (*n* = 6) of the patients had disease at stage I, followed by stage II (*n* = 11, 46%), stage III (*n* = 3, 13%), and stage IV (*n* = 4, 17%) disease. Histology analysis revealed that cases varied from adenocarcinoma to invasive CC, and squamous cell carcinoma was the most common (79%). The stages of CC were diagnosed using imaging-based methods (computed tomography and magnetic resonance imaging). Most of the patients were treated with cisplatin-based chemotherapy and radiation therapy, and the patients with small cell neuroendocrine carcinoma were treated with combination of cisplatin, paclitaxel, and bevacizumab. The radiation therapy regime was mainly administered to the pelvic site with 54Gy/30fx followed by ICR (Intracavity radation) 24Gy/6fx.

**Table 3:** Patient characteristics

Entire cohort	24
Histology	
Squamous cell carcinoma	19 (79%)
Endocervical adenocarcinoma	2 (8%)
Small cell neuroendocrine carcinoma	1 (4%)
Low-grade squamous intraepithelial neoplasia	1 (4%)
Invasive cervical cancer	1 (4%)
Pathogenic stage	
Stage I	6 (25%)
Stage II	11 (46%)
Stage III	3 (13%)
Stage IV	4 (17%)

### Genomic alterations in CC patients

The initial study was conducted by screening for overall genetic variation in patients with CC using our NGS panel. To explore the profiles of molecular variants, we analyzed cfDNA and PBMC that were extracted from the blood of 24 CC patients.

**Fig. 2:**
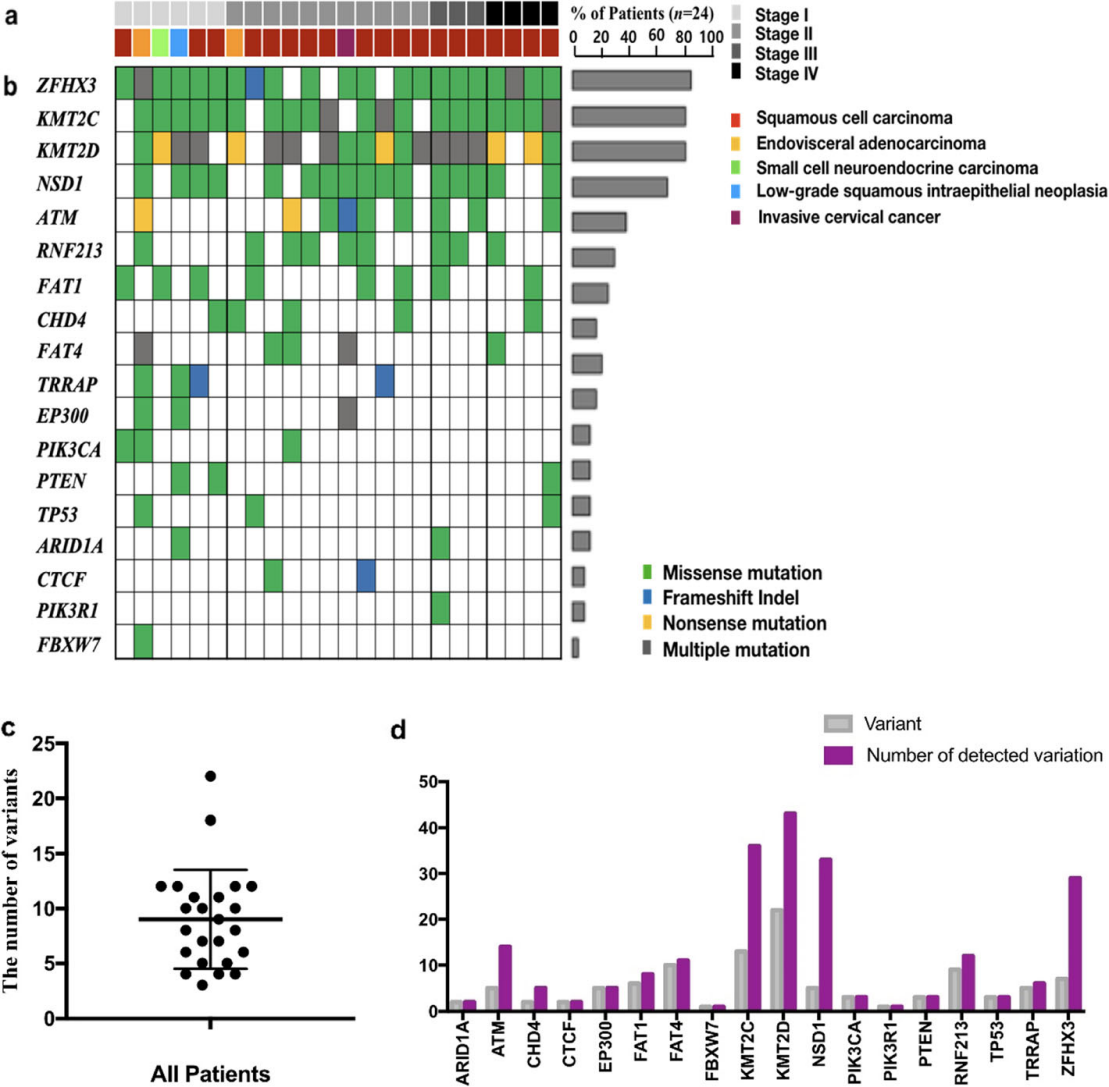
Somatic alterations in CC. **a**. Stages and the type of CC histology are represented. **b**. The Genetic variation panel lists the patient-specific variations, as well as the sequence of these variations. **c**. Dot plot indicates the number of variants in CC patients. d. Gray bar graphs show information for all gene variants, and purple bar graphs show the number of amplicons of gene variants detected in all patients

Twenty-four CC patients were sorted by different cancer stages and histology features (Fig. 2a and Supplemenatary Table 1). All patients with stages III and IV had the homogenous histology type as squamous cell carcinoma. Three patients (among the six patients with stage I disease) showed the same histology. Among the 24 genes in the list, alterations were found in 18 genes (75%) and no mutations were found in the remaining six genes (*BCOR*, *CTNNB1*, *FGFR2*, *OR14K1*, *POLE*, and *KRAS*). Genetic mutations in *ZFHX3*, *KMT2C/D*, and *NDS1* were detected in 20 (83%), 19 (79%), and 16 (67%) women with CC, respectively (Fig. 2b). These genes have been reported as tumor suppressors and are prevalent in other cancer-related diseases [18–23]. According to the data published by TCGA, *PIK3CA* (26%), *EP300* (11%), *FXBW7* (11%), and *PTEN* (8%) are the common genetic variants in CC [14]. However, our analysis showed that alterations within these genes occurred in 12.5, 12.5, 4, and 8% of the cases, respectively. Out of all the variant types, the missense mutations (24%) accounted for the largest number of variant types. Mutation patterns with two or more mutation types (such as missense and frameshift) were found in 15 patients. Frameshift insertions and deletions were found in only five patients. Overall, at least three genetic variants were found in all patients, with an average of 9 mutations per patient (Fig. 2c). The largest number of mutations was 22 variants in one patient. The total number of distinct mutations was 217 across all patients. After analyzing all variants of each gene, the most commonly detected variations were located in *KMT2D* [23], followed by the *KMT2C* [13], *FAT4* [10], *RNF213* [9], and *ZFHX3* [7] variants (Fig. 2d). Most mutations in cancer suppressor genes were evenly detected across all stages of cancer, whereas cancer driver gene variants were found mainly in the early stages of cancer (stage I and II).

### Variant allele frequency for patient monitoring

Among the 24 patients, 4 patients who agreed to monitor were selected. All assigned patients had been diagnosed with CC and showed the same general squamous cell carcinoma histology. The chemotherapy regimens were CDDP #6 (cisplatin) and radiotherapy was operated on pelvis with 54Gy/30fx followed by ICR 24Gy/6fx.

**Fig. 3:**
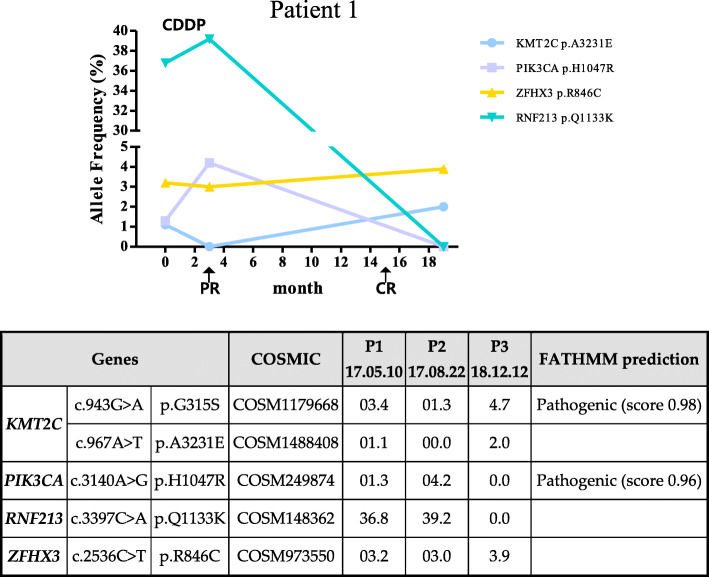
Patient specific features of tumor suppressor gene mutation. PR: partial response. CR: complete response. P1: Period 1, P2: Period 2, P3: Period 3

Patient 1 was 74 years old and was confirmed to have squamous cell carcinoma (stage IV) by surgical pathological examination. The follow-up period was approximately 19 months. The CC panel analysis revealed a total of four gene mutations (Fig. 3). In addition to *KMT2C* and *ZFHX3* mutations found in most patients, *PIK3CA* and *RNF213* mutations were also detected, and *RNF213* mutations changed over 18 months of the examination. Initial test findings revealed that the uterine cervix had an intense increment in mass and was approximately 5 cm in size stained with fluorodeoxyglucose (FDG) and may have been invaded into the bladder posterior wall. The patient was treated with CDDP for approximately 2.5 months. After undergoing chemotherapy (P2), a therapeutic effect was confirmed (partial response; PR). The *KMT2C* and *PIKC3CA* mutations, which were elevated in number in the early stages of chemotherapy, declined over time. By the time of the third examination, no *PIK3CA* and *RNF213* mutations were detected. The score for the FATHMM (http://fathmm.biocompute.org.uk) pathological prediction is 0.98 for *KMT2C* and 0.96 for *PIK3CA*, respectively. The third examination showed no residues from previous tumor observation (complete response; CR).

**Fig. 4:**
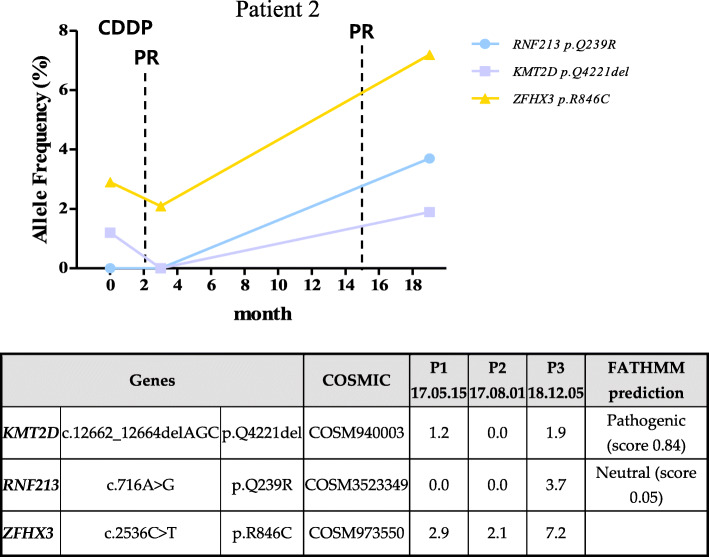
Patient specific features of tumor suppressor gene mutation

Patient 2 was 56 years old and was confirmed to have squamous cell carcinoma (stage II) by surgical pathological examination. The follow-up period was approximately 19 months. The patient did not undergo any further surgery, and the imaging findings revealed FDG-avid malignancy in the uterine cervix with extension into the uterine body and fundus. A total of three genetic mutations were found, which tended to decrease the allele frequencies overall with initial chemotherapy (Fig. 4). The variation of allele frequency in RNF213, which did not appear in the first and second examinations, until it was found at a 3.7% AF during the third examination. The mutation of *KMT2D* gene, which is considered as pathologic (0.84) according to the FATHMM prediction, was detected in the first screening but disappeared in the second screening and then reappeared in the third screening. The genetic variation of *ZFHX3* was found to decrease in the early phase of chemotherapy (2.1%), but increased over time (7.2%). At the third clinical examination, a mass distinct from the cervix was detected, and a slight thickening across the endometrium was also detected; otherwise, no measurable enlarged lymph nodes or fluid collections were observed around the lesion. As a result, although the CC size did not increase significantly, this patient was diagnosed with partial response to chemotherapy, due to other factors around cervix lesion site.

**Fig. 5:**
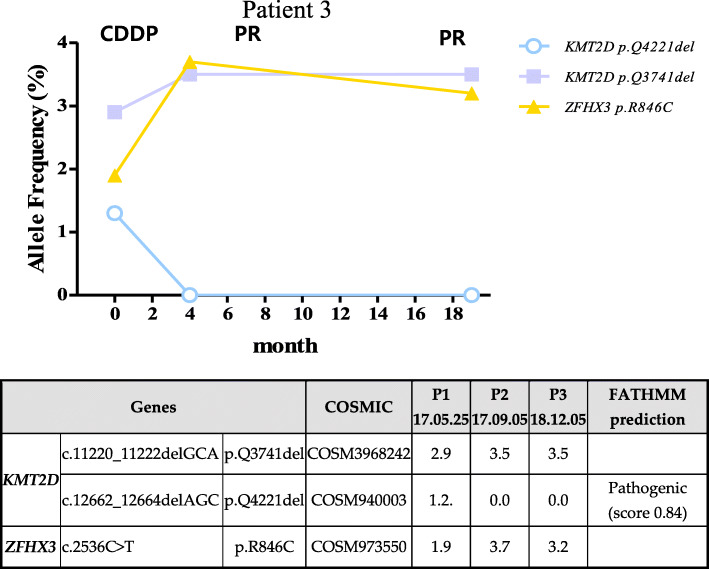
Patient specific features of tumor suppressor gene mutation

Patient 3 was 48 years old and had squamous cell carcinoma type CC (stage II) which tended to be keratinized. The patient had mutations in two genes (*KMT2D* and *ZFHX3*); the *KMT2D* mutation, which is considered as pathogenic (score 0.84), disappeared by the second and third screening (Fig. 5). The initial screening showed localized metastasis to the lymph node (LN) region of the uterine cervix and multiple myomas in the uterus. The treatment of this patient involved approximately 2 months of chemotherapy. Ten months later, the third examination was performed. Examination revealed no lesion sites in the uterine cervix. The LN of approximately 1.3 cm was still visible but was decreased in size. Additionally, leiomyoma of less than 4 cm was observed in the uterus. However, several uterine leiomyoma and endometrial polyps were observed (PR).

**Fig. 6:**
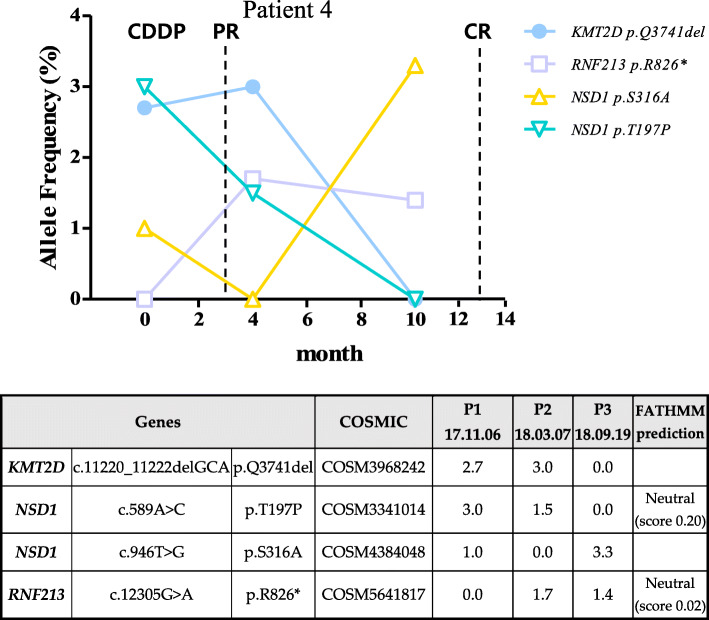
Patient specific features of tumor suppressor gene mutation

Patient 4 was 51 years old and had stage III cancer. The patient was followed up for approximately 13 months. The patient’ positron emission spectroscopy images showed an FDG-avid mass (SUVmax = 22.33) of a metabolic size of approximately 5 × 2.5 cm in the uterine cervix in the abdomen and pelvis. The metabolic length was approximately 5.5 cm, extending into the vagina and abutting the bladder base. Both external iliac LNs were up to 8 mm in diameter and showed FDG uptake. Other internal iliac LNs appeared to be small in size and did not show FDG uptake. In this patient, three major gene mutations *(KMT2D, NSD1* and *RNF213*) were found (Fig. 6). After treatment, the two of the three genetic variants disappeared. The *RNF213* gene mutation was not observed in the first screening, whereas its AF was found in the second and third screenings. After the third examination, lesions observed in the cervix and vagina anterior portions were not visible. There was no change in the sub centimeter-sized myomas in the uterine fundus. No significantly enlarged LN was observed in the pelvic cavity, and no abnormal fluid collection was observed. There were no abnormal findings in the metastases, urinary bladder, and rectum in the pelvic bone. In conclusion, a complete response was confirmed based on the difficulty in detecting the lesion site.

## Discussion

In the present study, we analyzed cfDNA from patients with CC by NGS using a customized panel of 24 cancer-related genes using the Ion Torrent system. The study was conducted to identify cfDNA mutations and explore their effectiveness in diagnosing and monitoring CC. There were three key challenges faced during this study. (i) All 24 subjects provided cancer-positive samples, however, we did not have non-patients samples to compare with. For a better understanding of the cancer-positive DNAs, samples from healthy donors must be included in cfDNA library preparation and sequencing, and cut-off values must also be validated more robustly for variant calling. (ii) In the analysis of assay specificity, there was excessive noise in variant calling. According to TCGA, there was an average of 4 mutations per Mbp. Although we minimized the technical errors and germline variants, there were approximately five mutations in 52 Mbp. Various data must be included to demonstrate the specificity of somatic variant calling. These data may include a list of specific variant locations and nucleotide changes across all samples. Other than established hotspot mutations, repeated variants across multiple samples may indicate technical errors. (iii), The detection sensitivity was limited. The levels of mutations in *PIK3CA*, *KRAS*, and *TP53*, the most relevant mutated genes in CC, were lower than expected. Because of the small positive cohort group (24 CC-positive patients), statistical analysis was difficult. Technical evaluation must be performed to further evaluate the assay sensitivity.

Recent studies have shown that the role of *KMT2C/D* gene is generally known to perform enhancer regulation by deposition of H3K4me1 in normal cells [24, 25] and a transcription regulator in cancer [26, 27]. Likewise the *KMT2C/D* gene, which may play an important role in cancer, is reported to have a frequency of up to 89% somatic mutation in esophageal squamous cell carcinoma (ESCC), medulloblastoma, follicular lymphoma, and diffuse large B-cell lymphoma patients [28]. More intriguingly, G. Paolo et al. showed that the frequency of *KMT2C/D* gene mutation was also notable in patients with histology of Cutaneous squamous cell carcinoma (SCC), head and neck SCC, lung SCC, esophageal SCC, and cervical SCC [29]. In similar to these studies, our results indicate that mutation rates of *ZFHX3*, *KMT2C*, *KMT2D*, *NSD1*, and *RNF213* genes have existed at a high frequency in CC patients in despite of the characteristics of these genetic mutations that have not been clearly identified in CC patients. The reason is that 79% of histologic subtype of CC in our cohort is consisted of squamous cell carcinoma (Table 1). Therefore, our findings on these genetic variations may be applicable to future studies of the molecular mechanism of cervical cancer.

In addition, *RNF213* mutation, which was employed as a monitoring marker for CC patients in our study, is currently little known about its function and role in cancer. *RNF213* is primarily known as E3 ubiquitin-protein ligase involved in angiogenesis and non-canonical signal pathway in vascular development. A preceding study is mainly focused on Moyamoya disease, and a cerebrovascular disease characterized by progressive bilateral stenosis of internal carotid arteries [30]. Therefore, the role of *RNF213* in cervical cancer is required for further investigation and must be validated as a prognostic factor to measure clinical outcomes during cervical cancer treatment.

We report several important aspects regarding the promising application of cfDNA for early diagnosis and monitoring of CC: (i) Gene mutation can serve as a prognostic biomarker for detecting CC by the profiling of the tumor suppressor and cancer driver genes. (ii) Mutations in tumor suppressor genes are prevalent in all stages of CC, and (iii) Chemotherapy and radiotherapy affect the allele frequency, which can be utilized for monitoring cancer. We also report the comprehensive mutation profile of CC samples. Notably, frequently mutated genes, such as *TP53* or *PIK3CA* in CC, were not predominantly identified in 24 Korean women. Interestingly, during the course of treatment of CC, we discovered that continuous observation of Tumor suppressor gene mutations could be employed to reveal the appropriate treatment modalities in patients. This approach of using liquid biopsy to detect the mutation pattern can be used in clinical practice. Although specific anticancer drugs for CC treatment have not yet been approved by the Food and Drug Administration, drugs prescribed for other carcinomas or radiation therapy can be used. Additionally, NGS technology, which can be explicitly used for the diagnosis of CC, needs to have a more accurate clinical specificity by minimizing false-positive diagnosis.

## Conclusion

CC is the cause of malignancy-related death among women. For the clinically usage in clinicians and patients parts, we developed the NGS CC panel. Through NGS analysis with blood samples in Korean women, the genetic variations in CC were found that are related to the genetic alteration result of TCGA and COSMIC data. Although we have some technical tasks to improve, we showed the advanced step of CC diagnosis with the NGS technology with blood. Our multifaceted approach to assessing genetic variations can be used for the diagnosis, monitoring, and further treatment of CC.

## Supplementary information

**Additional file 1: Supplementary Table 1.** The list of genetic variants at baseline.

## Data Availability

Not applicable.

## Abbreviations

AF: Allele Frequency
CC: Cervical Cancer
cfDNA: Cell-free DNA
ctDNA: Circulating tumor DNA
CR: Complete Response
FDG: Fluordeoxyglucose
HR-HPV: High-risk Human papillomavirus
ICR: Intracavity Radiation
LN: Lymph node
NGS: Next-Generation-Sequencing
PBMC: Peripheral Blood Mononuclear Cell
PR: Partial Response
SCC: Squamous cell carcinoma
SNP: Single Nucleotide Polymorphism
TCGA: The Cancer Genome Atlas
